# Clinical utility of *TERT* promoter mutations and *ALK* rearrangement in thyroid cancer patients with a high prevalence of the *BRAF* V600E mutation

**DOI:** 10.1186/s13000-016-0458-6

**Published:** 2016-02-09

**Authors:** Ja Seong Bae, Yourha Kim, Sora Jeon, Se Hee Kim, Tae Jung Kim, Sohee Lee, Min-Hee Kim, Dong Jun Lim, Youn Soo Lee, Chan Kwon Jung

**Affiliations:** Department of Surgery, College of Medicine, The Catholic University of Korea, Seoul, 06591 Republic of Korea; Department of Hospital Pathology, Seoul St. Mary’s Hospital, College of Medicine, The Catholic University of Korea, 222 Banpodaero, Seocho-gu, Seoul 06591 Republic of Korea; Department of Biomedicine & Health Sciences, College of Medicine, The Catholic University of Korea, Seoul, 06591 Republic of Korea; Department of Internal Medicine, College of Medicine, The Catholic University of Korea, Seoul, 06591 Republic of Korea

**Keywords:** Thyroid cancer, BRAF V600E, Telomerase reverse transcriptase, Anaplastic lymphoma kinase, Iodine-131

## Abstract

**Background:**

Mutations in the *TERT* promoter, *ALK* rearrangement, and the *BRAF* V600E mutation are associated with aggressive clinicopathologic features in thyroid cancers. However, little is known about the impact of *TERT* promoter mutations and *ALK* rearrangement in thyroid cancer patients with a high prevalence of *BRAF* mutations.

**Methods:**

We performed Sanger sequencing to detect *BRA*F V600E and *TERT* promoter mutations and both immunohistochemistry and fluorescence in situ hybridization to identify *ALK* rearrangement on 243 thyroid cancers.

**Results:**

*TERT* promoter mutations were not present in 192 well-differentiated thyroid carcinomas (WDTC) without distant metastasis or in 9 medullary carcinomas. However, the mutations did occur in 40 % (12/30) of WDTC with distant metastasis, 29 % (2/7) of poorly differentiated carcinomas and 60 % (3/5) of anaplastic carcinomas. *ALK* rearrangement was not present in all thyroid cancers. The *BRAF* V600E mutation was more frequently found in WDTC without distant metastasis than in WDTC with distant metastasis (*p* = 0.007). In the cohort of WDTC with distant metastasis, patients with wild-type *BRAF* and *TERT* promoter had a significantly higher response rate after radioiodine therapy (*p* = 0.024), whereas the *BRAF* V600E mutation was significantly correlated with progressive disease (*p =* 0.025).

**Conclusions:**

The *TERT* promoter mutation is an independent predictor for distant metastasis of WDTC, but *ALK* testing is not useful for clinical decision-making in Korean patients with a high prevalence of the *BRAF* V600E mutation. Radioiodine therapy for distant metastasis of WDTC is most effective in patients without *BRAF* V600E and *TERT* promoter mutations.

## Background

Thyroid cancer is the most common type of endocrine tumor, with an incidence that has significantly increased in the last few decades [1, 2]. Although well-differentiated thyroid carcinoma (WDTC) is one of the most curable of all cancers, approximately 10–20 % of patients with WDTC suffer from disease recurrence after surgery, and some eventually die from the disease [3–5]. Various risk stratification methods have been used for the proper management of patients with WDTC; however, none are completely accurate [6].

Molecular biomarkers have been used as an adjunct diagnostic marker of thyroid cancer and a predictor of patient prognosis [7, 8]. The *BRAF* V600E mutation is the most common mutation in thyroid cancer, particularly in papillary thyroid carcinoma (PTC), and plays an important role in tumorigenesis and progression [9–14]. In Korea, PTC comprises 97.3 % of all thyroid cancers according to new data from the 2014 annual report of cancer statistics in Korea (http://www.cancer.go.kr/). The *BRAF* V600E mutation is highly prevalent in Korean PTC patients [11]. Currently, there is controversy regarding whether the *BRAF* V600E mutation is associated with poor prognosis and aggressive clinicopathologic features in Korean PTC patients; therefore, additional prognostic biomarkers to predict a more aggressive disease are needed [9, 15–18].

**Fig. 1:**
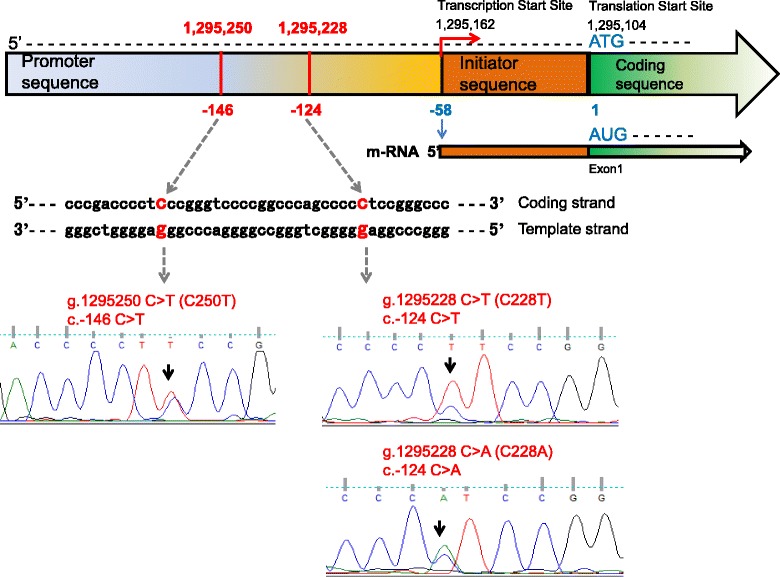
Structure of the wild-type *TERT* gene and representative sequencing electropherograms of the genomic DNA of the *TERT* promoter. The g.1295228 C > T (C228T) and g.1295250 C > T (C250T) mutations within the *TERT* promoter gene result in a cytosine-to thymine transition at 124 bp (c.-124C > T) and 146 bp (c.-146C > T) upstream of the ATG start codon, respectively. g.1295228 C > A (C228A) is a cytosine-to adenine transition at the 1 295 228 position of chr5

Somatic mutations of the promoter region of the *TERT* gene have been reported in various cancers, including thyroid cancers, but are not found in normal cells [19–23]. The frequent cytosine-to-thymine transition of the *TERT* promoter region occurs at the following positions of chr5: 1 295 228 (C228T) and 1 295 250 (C250T), which correspond to nucleotide changes -124 bp (c.-124C > T) and -146 bp (c.-146C > T) upstream from the ATG start site, respectively (Fig. 1) [19–23]. These *TERT* promoter mutations stimulate *TERT* transcriptional activity in cancer cells [19–23]. In thyroid cancers, *TERT* promoter mutations were predominantly found in more aggressive disease, such as tall cell variant PTC, widely invasive follicular thyroid carcinoma (FTC), poorly differentiated carcinoma, and anaplastic carcinoma [13, 18, 21, 24, 25].

*ALK* gene rearrangements have recently been identified in thyroid cancer [26–30]. *EML4*, *STRN*, *TFG*, and *GTF2IRD1* have been reported as *ALK* fusion partners [27, 28, 30–32]. The prevalence of *ALK*-rearranged PTCs has been reported to be up to 2.2 %, although the number of study cases is limited [26]. A previous study reported that *ALK* rearrangements were more frequently found in aggressive thyroid cancer, while another study found mutations only in unselected consecutive PTC cases and not in aggressive disease, such as PTCs with distant metastasis, poorly differentiated carcinomas and anaplastic carcinomas [26, 28].

We aimed to investigate the prevalence of *TERT* promoter and *ALK* mutations in thyroid cancer patients with a high prevalence of the *BRAF* V600E mutation and their potential contribution for the risk stratification of these patients.

## Methods

### Patients

We retrospectively enrolled 243 patients who underwent thyroid surgery for thyroid cancer at Seoul St. Mary’s Hospital of The Catholic University of Korea with the approval of the Institutional Review Board. Informed consent was obtained from every patient. The thyroid cancers studied included 192 consecutive WDTCs without distant metastasis (consisting of 127 classic PTCs, 11 classic PTCs with tall cell features, 9 encapsulated follicular variant PTCs, 7 infiltrative follicular variant PTCs, 16 tall cell variant PTCs, 1 oncocytic PTC, 1 Warthin-like PTC, and 20 minimally invasive FTCs), 30 consecutive WDTCs with distant metastasis (consisting of 14 classic PTCs, 4 classic PTCs with tall cell features, 1 encapsulated follicular variant PTC, 1 macrofollicular variant PTC, 5 tall cell variant PTCs, 1 columnar cell variant PTC, 1 diffuse sclerosing variant PTC, 2 minimally invasive FTCs, and 1 widely invasive FTC), 7 poorly differentiated carcinomas, 5 anaplastic carcinomas, and 9 medullary carcinomas. PTC was defined as a classic type with tall cell features if it consisted of less than 50 % tall cells and as a tall cell variant if it consisted of 50 % or more tall cells [33].

### Mutational analyses for *BRAF* and *TERT* promoter mutations

Genomic DNA was isolated from manually dissected 10-μm thick paraffin-embedded tissue sections using the RecoverAll™ Total Nucleic Acid Isolation Kit (Life Technologies, Carlsbad, CA, USA). Sanger sequencing was performed to detect the presence of *BRAF* V600E and *TERT* promoter mutations. Exon 15 of the *BRAF* gene was PCR-amplified as previously reported using the following forward primer (5′-TCATAATGCTTGCTCTGATAGGA-3′) and reverse primer (5′-GGCCAAAAATTTAATCAGTGGA-3′), resulting in a 224 bp PCR product [11, 33, 34]. A 193 bp fragment of the *TERT* promoter was amplified by PCR as previously reported using the following forward primer (5′-CACCCGTCCTGCCCCTTCACCTT-3′) and reverse primer (5′-GGCTTCCCACGTGCGCAGCAGGA-3′) [35]. All *TERT* promoter mutations were confirmed using another previously reported primer set that included the following forward primer (5′-AGTGGATTCGCGGGCACAGA-3′) and reverse primer (5′-CAGCGCTGCCTGAAACTC-3′) and resulted in a 235 bp PCR product [21].

### Immunohistochemistry for *ALK* overexpression

Immunohistochemistry was performed on paraffin-embedded whole tissue sections of surgical specimens using the ALK antibody (clone p80, Novocastra Laboratories Ltd., Newcastle upon Tyne, UK) and the Polink-2 HRP plus anti-rabbit DAB detection kit (GBI Labs, Mukilteo, WA, USA). As a positive control, we used paraffin-embedded tissue sections from two lung adenocarcinomas with previously confirmed *ALK* rearrangement by fluorescence in situ hybridization (FISH).

### FISH for *ALK* rearrangement

We performed FISH to detect *ALK* rearrangement using a ZytoLight SPEC ALK Dual Color Break Apart Probe and Kit (ZytoVision GmbH, Bremerhaven, Germany) according to the manufacturer’s protocol [29]. The positive criterion for *ALK* rearrangement was defined as > 15 % of split signal separation and/or isolated red signal in at least 100 tumor cells as previously described [26, 29].

### Evaluation of response to radioiodine therapy

All 30 WDTC patients with distant metastasis underwent radioactive iodine (RAI) therapy. The response to RAI ablation was evaluated with a whole body iodine -131 scan, evaluation of serum thyroglobulin levels, and a computerized tomography scan. Clinical outcomes to RAI therapy were classified as complete remission (CR), partial response (PR), stable disease (SD), and progressive disease (PD) according to previously described criteria [36].

### Statistical analysis

The Pearson’s chi-square test or Fisher’s exact test was used to assess the relationship between two nominal variables. The Student’s *t*-test and Mann–Whitney test were used to compare two different groups of continuous parametric or nonparametric data, respectively. For the multivariate analysis, parameters that were significant at *p* < 0.25 in the univariate analysis were included in a multiple logistic regression test. Two-sided tests with *p* < 0.05 were considered to be statistically significant. Statistical analysis was performed with SPSS ver. 21.0 software (SPSS Inc., Chicago, IL, USA) and SAS ver. 9.3 software (SAS Institute Inc., Cary, NC, USA).

### Meta-analysis of the proportion of *TERT* promoter mutations

We searched the literature for *TERT* promoter mutations in thyroid cancer using PubMed and Google up to November 2015, and selected eligible articles. We then conducted a meta-analysis of the proportion of *TERT* promoter mutations according to the histologic types of thyroid cancers. Cochran Q test and I^2^ values were employed to assess statistical heterogeneity among studies. If significant heterogeneity was observed (*p* < 0.10 or I^2^ > 50 %), the random effect model was used for meta-analysis. Otherwise, we used a fixed-effect model for the meta-analysis. Meta-analyses were performed using done using MedCalc version 13.0.2 software (MedCalc, Ostend, Belgium).

## Results

### Prevalence of *TERT* promoter mutations, the *BRAF* V600E mutation, and *ALK* rearrangement in thyroid cancers

*TERT* promoter mutations were found in 12 (40 %) of 30 WDTCs with distant metastasis, 2 (29 %) of 7 poorly differentiated carcinomas, and 3 (60 %) of 5 anaplastic carcinomas. However, no such mutations were present in the 192 WDTCs without distant metastasis or the 9 medullar carcinomas (Table 1). Among *TERT* promoter mutations, the most common type was C228T (76 %), followed by C250T (18 %) and C250A (6 %) (Table 1) (Fig. 1). Among 12 WDTCs with *TERT* promoter mutations, the most frequent histologic subtype was the tall cell variant of PTC (Table 1).

**Table 1:** *TERT* promoter mutations, *BRAF* V600E mutation and *ALK* rearrangement in 243 Korean patients with thyroid cancer

	Patient	*TERT* promoter mutation				*BRAF* V600E	*ALK* rearrangement
		C228T	C250A	C250T	Overall		
WDTC without distant metastasis	192	0	0	0	0	142 (74 %)	0
PTC, classic	127	0	0	0	0	110 (87 %)	0
PTC, classic with TCF	11	0	0	0	0	10 (91 %)	0
PTC, EFV	9	0	0	0	0	1 (11 %)	0
PTC, IFV	7	0	0	0	0	5 (71 %)	0
PTC, tall cell	16	0	0	0	0	15 (94 %)	0
PTC, oncocytic	1	0	0	0	0	1 (100 %)	0
PTC, Warthin-like	1	0	0	0	0	0	0
FTC, minimally invasive	20	0	0	0	0	0	0
WDTC with distant metastasis	30	10 (33 %)	0	2 (7 %)	12 (40 %)	15 (50 %)	0
PTC, classic	14	3 (21 %)	0	0	3 (21 %)	7 (50 %)	0
PTC, classic with TCF	4	2 (50 %)	0	1 (25 %)	3 (75 %)	4 (100 %)	0
PTC, EFV	1	0	0	0	0	0	0
PTC, macrofollicular	1	0	0	0	0	0	0
PTC, tall cell	5	4 (80 %)	0	0	4 (80 %)	3 (60 %)	0
PTC, columnar cell	1	0	0	1 (100 %)	1 (100 %)	1 (100 %)	0
PTC, diffuse sclerosing	1	0	0	0	0	0	0
FTC, minimally invasive	2	1 (50 %)	0	0	0	0	0
FTC, widely invasive	1	0	0	0	1(100 %)	0	0
Poorly differentiated carcinoma	7	1 (14 %)	1 (14 %)	0	2 (29 %)	1 (14 %)	0
Anaplastic carcinoma	5	2 (40 %)	0	1 (20 %)	3 (60 %)	4 (80 %)	0
Medullary carcinoma	9	0	0	0	0	0	0

*WDTC* well-differentiated thyroid carcinoma, *PTC* papillary thyroid carcinoma, *TCF* tall cell features, *EFV* encapsulated follicular variant, *IFV* infiltrative follicular variant, *FTC* follicular thyroid carcinoma

The *BRAF* V600E mutation was found in 142 (83 %) of 172 PTCs without distant metastasis, 15 (56 %) of 27 PTCs with distant metastasis, 1 (14 %) of 7 poorly differentiated carcinomas and 4 (80 %) of 5 anaplastic carcinomas (Table 1). However, the *BRAF* V600E mutation was not found in 23 FTCs and 9 medullary carcinomas.

None of the 243 thyroid cancers had positive ALK immunohistochemistry or ALK break apart FISH (Table 1).

### Relationship between *TERT* promoter mutations and clinicopathologic features of WDTCs

In 222 patients with WDTC, the presence of *TERT* promoter mutations was associated with older age (*p =* 0.017), larger tumor size (*p =* 0.043), aggressive histologic subtypes (*p* < 0.001), advanced pathologic T stage (*p* = 0.014), extrathyroidal extension (*p =* 0.035), lymph node metastasis (*p =* 0.011), lateral lymph node metastasis (*p* < 0.001), distant metastasis (*p* < 0.001), and advanced AJCC stage (*p* < 0.001) (Table 2). There was no association between *TERT* promoter mutations and the *BRAF* V600E mutation (Table 2).

**Table 2:** Association between *TERT* promoter mutations and clinicopathologic features in 222 patients with well-differentiated thyroid carcinoma

	*TERT* promoter mutations		
	Absent (*n* = 210)	Present (*n* = 12)	*p*-value
Age (mean years)	45.5 ± 13.3	55.0 ± 11.8	0.017
Gender			
Female	164 (94.8 %)	9 (5.2 %)	0.801
Male	46 (93.9 %)	3 (6.1 %)	
Tumor size (mean mm)	14.8 ± 12.5	31.9 ± 22.9	0.043
Histologic types			
Aggressive variant^a)^	18 (75.0 %)	6 (25.0 %)	<0.001
Less-aggressive variant	192 (97.0 %)	6 (3.0 %)	
Pathologic T stage			
pT 1–2	97 (99.0 %)	1 (1.0 %)	0.014
pT 3–4	113 (91.1 %)	11 (8.9 %)	
Extrathyroidal extension			
Absent	105 (98.1 %)	2 (1.9 %)	0.035
Present	105 (91.3 %)	10 (8.7 %)	
Pathologic N stage			
pN0	105 (99.1 %)	1 (0.9 %)	0.011
pN1	105 (91.3 %)	10 (8.7 %)	
Lateral lymph node metastasis			
Absent	166 (98.8 %)	2 (1.2 %)	<0.001
Present	43 (82.7 %)	9 (17.3 %)	
Distant metastasis			
Absent	192 (100 %)	0	<0.001
Present	18 (60.0 %)	12 (40 %)	
AJCC stage			
I-II	120 (100 %)	0	<0.001
III-IV	90 (88.2 %)	12 (11.8 %)	
*BRAF* V600E mutation			
Absent	62 (95.4 %)	3 (4.6 %)	0.738
Present	148 (94.3 %)	9 (5.7 %)	

^a)^Aggressive variant includes 21 tall cell, 1 columnar cell, and 1 diffuse sclerosing variant of papillary carcinoma and 1 widely invasive follicular carcinoma

### Relationship between clinicopathologic and molecular features and distant metastases of WDTCs

The mean follow-up period of the patients with WDTC was 36.1 months. In 14 patients, distant metastases were found within 6 months of first diagnosis. Distant metastases occurred in the lung (*n* = 24), bone (*n* = 3), lung and bone (*n* = 2), and brain (*n* = 1). Distant metastasis was associated with larger tumor size (*p =* 0.001), aggressive histologic subtype (*p =* 0.003), advanced pT stage (*p* < 0.001), extrathyroidal extension (*p =* 0.001), lymph node metastasis (*p* < 0.001), lateral lymph node metastasis (*p* < 0.001) and the *TERT* promoter mutation (*p* < 0.001). However, the *BRAF* V600E mutation was inversely associated with distant metastasis (*p =* 0.007).

In the multivariate analysis, the odds ratio (OR) for distant metastasis of WDTC in patients harboring tumors with *TERT* promoter mutations and lateral lymph node metastases was 155.298 (95 % confidence interval (CI) 3.362–999.990) and 11.159 (95 % CI 1.902–65.461), respectively (Table 3). The OR for distant metastases of WDTC in patients harboring tumors with the *BRAF* V600E mutation was 0.083 (95 % CI 0.021–0.327) (Table 3).

**Table 3:** Multivariate analysis of factors affecting distant metastasis

	Odds Ratio	95 % CI	*p*-value
Age	1.046	0.999–1.095	0.054
Gender	0.688	0.197–2.401	0.557
Tumor size	1.04	0.999–1.095	0.083
Histologic type	0.816	0.182–3.66	0.790
Pathologic T stage	0.142	0.005–4.016	0.252
Extrathyroidal extension	19.535	0.618–617.017	0.091
Pathologic N stage	0.922	0.141–6.053	0.933
Lateral lymph node metastasis	11.159	1.902–65.461	0.008
*TERT* promoter mutation	155.298	3.362–999.990	0.009
*BRAF* V600E mutation	0.083	0.021–0.327	<0.001

### Impact of molecular genotypes on response of metastatic WDTCs to RAI therapy

**Fig. 2:**
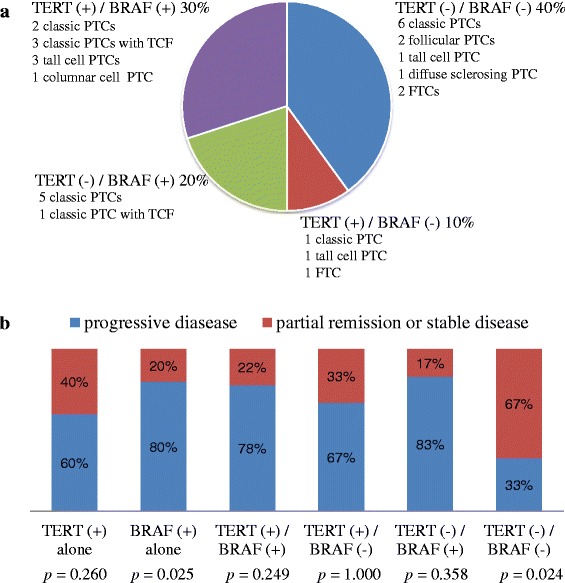
Molecular genotypes and radioactive iodine therapy. **a** Molecular genotypes of 30 well-differentiated thyroid carcinomas with distant metastases based on *TERT* promoter mutations and *BRAF* V600E. **b** Structural response of distant metastatic disease to radioactive iodine therapy classified by molecular genotypes in 30 well-differentiated thyroid carcinoma patients with distant metastasis. PTC, papillary thyroid carcinoma; TCF, tall cell features; FTC, follicular thyroid carcinoma; TERT, *TERT* promoter mutations; BRAF, *BRAF* V600E; (+), mutation positive; (-), mutation negative

Of the 30 WDTCs with distant metastasis, 9 (30 %) had coexisting *BRAF* V600E and *TERT* promoter mutations and 12 (40 %), including follicular and diffuse sclerosing variants of PTC, had no mutations (Fig. 2a).

The structural response of distant metastatic disease to RAI was evaluated at least 6 months after RAI therapy. Of the 30 WDTC patients with distant metastasis, six (20 %) patients had PR and six (20 %) had SD after RAI ablation whereas none achieved CR and 18 (60 %) had PD. There was a significant correlation between tumors with the *BRAF* V600E mutation alone and the progression of distant metastatic disease after RAI therapy (*p =* 0.025), but *TERT* promoter mutations alone were not associated with PD (Fig. 2b). PR or SD after RAI therapy was significantly more likely in patients with wild-type *BRAF* and *TERT* promoter genes (*p =* 0.024) (Fig. 2b). However, other combinations of genetic mutations were not correlated with RAI response.

### Meta-analysis of *TERT* promoter mutation prevalence in thyroid cancer

**Fig. 3:**
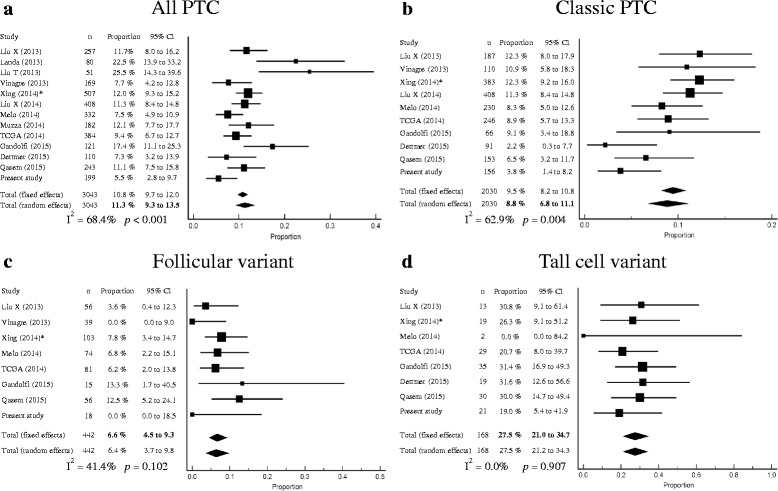
Forest plots of the meta-analysis for the prevalence of *TERT* promoter mutations in all papillary thyroid carcinomas (PTC), classic PTC (**b**), follicular variant of PTC (**c**), and tall cell variant of PTC (**d**). *Only *TERT* C228T was examined. TCGA, The Cancer Genome Atlas

**Fig. 4:**
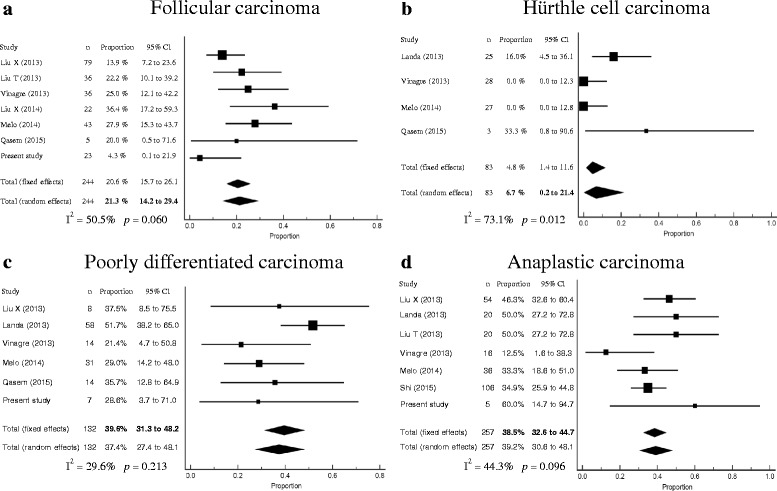
Forest plots of the meta-analysis for the prevalence of *TERT* promoter mutations in follicular thyroid carcinomas (**a**), Hürthle cell carcinoma (**b**), poorly differentiated carcinoma (**c**), and anaplastic carcinoma (**d**)

Our study and 13 articles were included for the meta-analysis of *TERT* promoter mutation prevalence in various thyroid cancers [17, 18, 21, 24, 25, 32, 37–43]. Significant heterogeneity was found in classic PTC, FTC, Hürthle cell carcinoma, and anaplastic carcinoma among the studies (Figs. 3 and 4). The mean frequencies of *TERT* promoter mutations in PTC, conventional FTC, Hürthle cell carcinoma, poorly differentiated carcinoma and anaplastic carcinoma were 11.3 % (95 % CI 9.3–13.5), 21.3 % (95 % CI 14.2–29.4), 6.7 % (95 % CI 0.2–21.4), 39.6 % (95 % CI 31.3–48.2), and 38.5 % (95 % CI 32.6–44.7), respectively (Figs. 3 and 4). When PTCs were stratified by histologic subtype, mean frequencies of *TERT* promoter mutations in classic, follicular, and tall cell variants were 8.8 % (95 % CI 6.8–11.1), 6.6 % (95 % CI 4.5–9.3), 27.5 % (95 % CI 21.0–34.7), respectively (Fig. 3). *TERT* promoter mutations were not found in a total of 132 medullary carcinoma patients including our case series [17, 21, 24, 25, 41].

## Discussion

We found that *TERT* promoter mutations are prevalent in aggressive thyroid cancers and are associated with distant metastasis of WDTCs in Korean patients with a high prevalence of the *BRAF* V600E mutation. When we examined *TERT* promoter mutations in a consecutive series of 192 WDTC patients who had no distant metastasis during the follow-up period, none carried the mutation. However, *TERT* promoter mutations were found in 40 % of WDTC patients with distant metastasis. In all 222 WDTC patients, the overall prevalence of *TERT* promoter mutations was 5.4 %. These results are lower than those reported in other countries. The prevalence of *TERT* promoter mutations reported in the literature ranged from 7.3 to 25.5 % in PTC and from 4.3 to 36.4 % in FTC [17, 18, 21, 24, 25, 32, 37–43].

In our study, *TERT* promoter mutations were associated with older age, larger tumors, higher stage and distant metastases in WDTCs. These findings are consistent with those of previous reports indicating that *TERT* promoter mutations are associated with aggressive clinical behavior [21, 24]. In the stratified meta-analysis by histologic subtype of PTC, we found that the prevalence of *TERT* promoter mutations was correlated with the degree of tumor aggressiveness. The tall cell variant of PTC exhibits more aggressive behavior than classic PTC [27, 33], while clinical features of the follicular variant of PTC are between classic PTC and FTC [44]. The *TERT* promoter mutations were most frequently found in tall cell variant (27.5 %, 95 % CI 21.0–34.7), followed by classic PTC (8.8 %, 95 % CI 6.8–11.1) and follicular variant (6.6 %, 95 % CI 4.5–9.3). These results are consistent with findings of present study.

Many studies have shown the role of *BRAF* V600E in advanced clinical stage and distant metastasis of PTC [7, 45]. In contrast, we found that the *BRAF* V600E status was inversely correlated with the rate of distant metastasis in WDTCs. This contradiction may be related to case selection bias. Of 30 metastatic tumors enrolled in our study, 20 % included follicular and diffuse sclerosing variants of PTC and FTCs, which were all negative for the *BRAF* V600E mutation. It is well-known that the incidence of *BRAF* V600E is very low in the follicular and diffuse sclerosing variants of PTC, and no FTCs have the *BRAF* V600E mutation [11, 34, 46].

In our study, the *BRAF* V600E mutation was significantly associated with low response rate of metastatic WDTCs to RAI therapy. These results are consistent with previous studies that have demonstrated high frequency of *BRAF*V600E mutation in RAI-refractory metastatic thyroid cancers [1, 47]. However, there was no significant effect of *TERT* promoter mutations on distant metastasis of WDTCs. The most likely mechanism of resistance to RAI therapy is the impaired iodide-handling machinery in metastatic thyroid cancer [1]. Many studies have reported that *BRAF*V600E mutation reduces the expression of thyroid iodine-handling genes (sodium iodide symporter, thyroid-stimulating hormone receptor, thyroglobulin, and thyroperoxidase) in thyroid cancer [1, 47, 48]. However, mechanism underlying the RAI therapy resistance associated with *TERT* promoter mutations remains uncertain. Xing et al reported that coexisting *BRAF* V600E and *TERT* C228T mutations defined the most aggressive subgroup of PTC when analyzed in terms of clinicopathologic features, tumor recurrence and disease-free survival rate [18]. We did not observe this trend in our study (data not shown).

Two *TERT* C228T and C250T mutations create consensus binding motifs for the E-twenty-six (ETS)/ternary complex transcription factor (TCF) and increase the transcriptional activity of the *TERT* promoter [19, 23]. *TERT* promoter mutations in thyroid cancer and glioma were associated with increased mRNA expression and telomerase activity [17, 49]. *BRAF* V600E and *TERT* promoter mutations can activate the mitogen-activated protein kinase (MAPK) signaling pathway in thyroid cancer [21]. In previous studies, *TERT* promoter mutations were more frequently found in *BRAF* V600E mutation-positive PTCs, suggesting an incremental and synergistic effect of the coexisting two mutations in tumorigenesis [18, 21]. In our study, the *TERT* promoter mutation status was not associated with the incidence of the *BRAF* V600E mutation. These discrepancies may be associated with ethnic differences given that there is a higher prevalence of the *BRAF* V600E mutation and lower occurrence of *TERT* promoter mutations in Korean patients than in Western patients. Therefore, our study results cannot be generalized to other populations.

We found no *TERT* promoter mutations in medullary carcinoma. This finding is consistent with previous reports [21, 24]. Moreover, *TERT* promoter mutations were not found in benign thyroid nodules, whereas they were more prevalent in poorly differentiated or anaplastic carcinomas than in WDTCs [21, 24]. Therefore, it is suggested that *TERT* promoter mutations are involved only in the tumorigenesis of follicular-cell derived thyroid cancers, particularly in aggressive subtypes, and may occur as a late molecular-genetic event that induces dedifferentiation of WDTCs [21].

*ALK* gene rearrangements are mutually exclusive with all other known thyroid cancer driver mutations and have been reported in up to 2.2 % of PTCs, 4 % of poorly differentiated carcinomas, and 4 % of anaplastic carcinomas [26, 28, 32]. In our study, *ALK* rearrangement was not identified in any thyroid cancers.

The main limitations of our study were the relatively small sample size of metastatic cancers and the short follow-up period. Although the analyses for disease recurrence and survival of patients were not available, we could evaluate the therapeutic response to RAI based on the distant metastatic disease genotypes. We report for the first time the clinical impact of *TERT* promoter mutations on thyroid cancers that occur in a *BRAF* V600E prevalent area.

## Conclusions

Our study demonstrated that Korean patients have a higher *BRAF* V600E prevalence and lower prevalence of the *TERT* promoter mutation and *ALK* rearrangement in thyroid cancers than do Western patients. *TERT* promoter mutation is associated with aggressive clinicopathologic features and is a strong predictor of distant metastasis of WDTC. In Korea, the *BRAF* V600E-negative WDTCs more frequently develop distant metastasis than *BRAF* V600E-positive tumors. When WDTC patients develop distant metastases, RAI therapy is most effective in patients without *BRAF* V600E and *TERT* promoter mutations. Further prospective evaluation that includes a larger sample size is needed.

## Abbreviations

CI: confidence interval
CR: complete remission
FISH: fluorescence in situ hybridization
FTC: follicular thyroid carcinoma
MAPK: mitogen-activated protein kinase
PD: progressive disease
PR: partial response
PTC: papillary thyroid carcinoma
RAI: radioactive iodine
SD: stable disease
WDTC: well-differentiated thyroid carcinoma
